# Communities of Arbuscular Mycorrhizal Fungi Detected in Forest Soil Are Spatially Heterogeneous but Do Not Vary throughout the Growing Season

**DOI:** 10.1371/journal.pone.0041938

**Published:** 2012-08-07

**Authors:** John Davison, Maarja Öpik, Martin Zobel, Martti Vasar, Madis Metsis, Mari Moora

**Affiliations:** 1 Department of Botany, Institute of Ecology and Earth Sciences, University of Tartu, Tartu, Estonia; 2 Centre for Biology of Integrated Systems, Tallinn University of Technology, Tallinn, Estonia; Argonne National Laboratory, United States of America

## Abstract

Despite the important ecosystem role played by arbuscular mycorrhizal fungi (AMF), little is known about spatial and temporal variation in soil AMF communities. We used pyrosequencing to characterise AMF communities in soil samples (n = 44) from a natural forest ecosystem. Fungal taxa were identified by BLAST matching of reads against the Maarj*AM* database of AMF SSU rRNA gene diversity. Sub-sampling within our dataset and experimental shortening of a set of long reads indicated that our approaches to taxonomic identification and diversity analysis were robust to variations in pyrosequencing read length and numbers of reads per sample. Different forest plots (each 10×10 m and separated from one another by 30 m) contained significantly different soil AMF communities, and the pairwise similarity of communities decreased with distance up to 50 m. However, there were no significant changes in community composition between different time points in the growing season (May-September). Spatial structure in soil AMF communities may be related to the heterogeneous vegetation of the natural forest study system, while the temporal stability of communities suggests that AMF in soil represent a fairly constant local species pool from which mycorrhizae form and disband during the season.

## Introduction

Arbuscular mycorrhizal fungi (AMF; phylum Glomeromycota; [1]) colonise the roots of most terrestrial plants, gaining plant-assimilated carbon while influencing mineral nutrient uptake, water relations and pathogen resistance in their hosts [2]. While functional aspects of plant-AMF interactions have been a major focus of research [3], [4], the essential ecosystem role played by AMF [5] and the commercial benefits of inoculum production [6] have stimulated interest in describing and explaining the distribution of AMF diversity. Two main approaches have been used to identify the AMF taxa present in ecosystems: (1) morphological and (rarely) molecular identification of fungal spores isolated from soil; and (2) molecular identification of the fungal structures (hyphae, arbuscules, vesicles) colonizing plant roots. However, these approaches overlook the non-spore AMF structures present in soil. More generally, diversity patterns in soil AMF communities have received little attention compared with those reflecting colonisation of plant roots.

AMF species have traditionally been described on the basis of the morphology and ontogeny of their spores. While identification of spores has also been widely used to characterise AMF communities in soil, e.g. [7], [8], sporulation is known to be a seasonal phenomenon that is dependent on the physiological status and identity of both fungus and host plant [9], [10]. A trap culture approach [11], used to trigger sporulation of the AMF present in a soil or root sample, can increase the quantity of spores used for identification. However, resulting AMF communities are also likely to differ from field spore communities [12], [13] since trap cultures may encourage the sporulation of different species than would associations with natural hosts in field conditions [13], [14].

Including into analysis the non-spore fungal structures formed within plant roots (arbuscules and vesicles) or in soil (auxiliary cells, branched absorbing structures) should provide more complete information about the presence and diversity of AMF taxa in ecosystems. Such structures cannot be precisely identified on a morphological basis (but see [15] for high taxonomic level identification), so PCR-based methods, often targeting nuclear rRNA genes, have been used to detect AMF *in planta* and in soil. Fungal DNA in plant roots derived from natural ecosystems has frequently been identified in this way (for reviews see [16]–[18]). Studies from temperate forests have revealed seasonal and habitat differences in intraradical AMF communities [19], [20], and host selectivity in plant-AMF interactions [21]–[23]. Moreover, high diversity of intraradical AMF has been recorded from forest ecosystems with low intensity management [20], [22], [24]. However, DNA-based studies of soil AMF communities have focused exclusively on semi-natural and anthropogenic systems, including grasslands [25]–[28]; agricultural ecosystems [29]–[39]; urban soil [40]; and semiarid shrubland [41], [42]. Describing AMF communities in natural soils, such as forest ecosystems, would provide an important context for observations of intraradical AMF and vascular plant diversity.

Since AMF DNA in soil incorporates both extraradical hyphae and spores, soil diversity measures could potentially describe the total AMF taxon pool, including actively functioning fungal taxa as well as dormant spores and taxa that have been active in the past. Hempel et al. [25] studied the molecular diversity of DNA extracted from the spores, roots and soil of the same samples and found that AMF community composition differed among fractions, with highest diversity recorded from the soil fraction.

Spatial and temporal variation in AMF communities has been relatively little studied using DNA-based techniques, and where it has been investigated, intraradical rather than soil AMF communities have generally been addressed. Thus, there is evidence to suggest that spatial variation exists in AMF communities derived from plant roots at the regional [43]–[45], local [19], [46] and plant neighbourhood scale (<2 m; [47]); though Öpik et al. [22] found no difference between plots in the same forest. In the only study to investigate spatial variability in soil, Mummey & Rillig [26] also noted autocorrelation in AMF communities at small (<1 m) scales. Meanwhile, temporal (seasonal) variation in AMF communities inhabiting plant roots has been recorded in several different systems [19], [20], [23], [48], [49]; while temporal dynamics of soil AMF communities are entirely unstudied using DNA based approaches. Spatial and temporal variation might result from species responses to soil physical and chemical properties, dispersal limitation, life history traits, host preference, and interactions between AMF species. Evidence from soils is required in order to clarify the importance of these factors and to gain greater insights into ecosystem functioning and diversity patterns in other organisms.

In this study we aimed to establish (i) the molecular diversity and (ii) spatial and temporal patterns of variation in soil AMF communities occurring at a primeval forest site, located in Järvselja, Estonia. We used pyrosequencing to characterise the fungal communities present in soil samples collected from spatially distinct plots over the course of the growing season (May – September).

## Methods

### Study Site

The study was conducted in Järvselja forest reserve in southeastern Estonia (58 17.916 N 27 15.744 E). The study area contains a mature mixed forest on gleyic soil with a herb-rich arbuscular mycorrhizal understorey (dominated by *Calamagrostis arundinacea* L. Roth, *Oxalis acetosella* L., *Hepatica nobilis* Mill., *Galeobdolon luteum* Huds.). The tree layer consists of Norway spruce (*Picea abies* L. H. Karst.) and deciduous tree species (most commonly *Acer platanoides* L., *Populus tremula* L., *Tilia cordata* Mill.) (see [50], [51] for descriptions of the area). Logging has been strictly prohibited at Järvselja since 1924, and the site is believed to have received minimal anthropogenic impact throughout history. No permits are required to carry out research on public land in Estonia, unless specified otherwise in legislation. No regulation applies to the Järvselja site.

### Sample Collection

**Figure 1:**
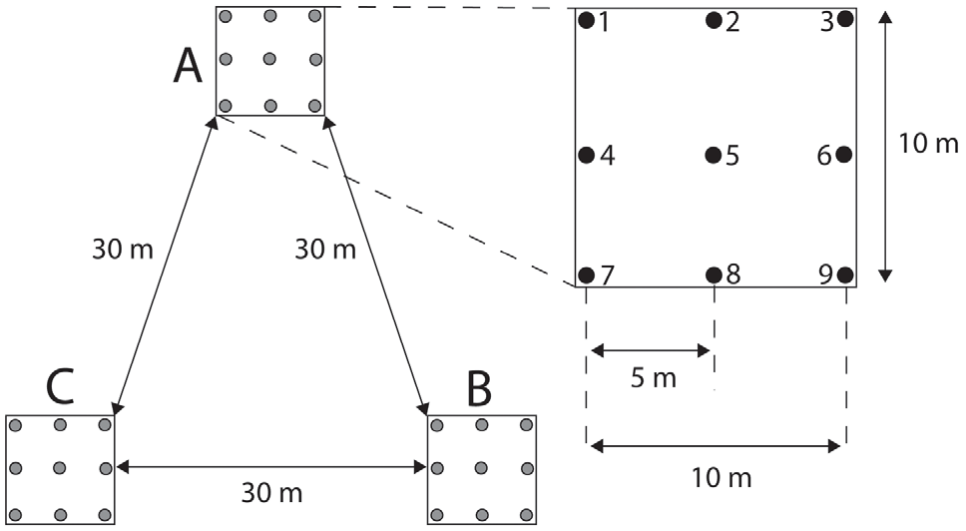
Sampling design used to study soil AMF communities in Järvselja forest reserve. Nine soil samples (SampleID 1–9) were collected in each of three 10×10 m plots (A, B and C). See Table 1 for further details of the sampling design.

Soil samples were collected from three 10×10 m plots located 30 m from one another in the forest (Figure 1). Plots were located in an area of forest that contained no obvious gradient in vegetation or physical characteristics (e.g. relief). Samples were collected from one plot (A) on 25^th^ May, 30^th^ June, 22^nd^ July and 3^rd^ September 2009, while the other two plots (B and C) were only sampled on 3^rd^ September 2009. Within each plot, soil was collected from nine points on a regularly-spaced sampling grid (Figure 1). During the repeat sampling of plot A, samples were taken from the same 10 cm patch around each grid point, with new samples taken from an undisturbed part of the small patch. Each soil sample consisted of 10 g of soil collected from the top 5 cm of soil beneath the litter layer. Samples were taken using sterile plastic spoons; a new spoon was used at each grid point. Sterile rubber gloves were also worn and changed at each grid point. Roots were removed from soil samples, which were then dried with silica gel and stored air-tight at room temperature.

### Molecular Analyses

DNA was extracted from 250 mg dried soil with PowerSoil® DNA Isolation Kit (MO BIO Laboratories, Inc., Carlsbad, CA, USA). Glomeromycota sequences were amplified from soil DNA extracts using the SSU rRNA gene primers NS31 and AML2 [52], [53], linked to sequencing primers A and B, respectively. While a number of different genes have been used to study AMF diversity, we targeted the SSU rRNA gene, since most data concerning the natural diversity of AMF have been obtained using this region [16], [17]. This gene consequently provided us with a larger comparative sequence dataset than would be available for any other genomic region [17]. In order to identify reads originating from different samples, we used a set of 8-base-pair barcodes designed following [54]. The barcode sequences were inserted between the A primer and NS31 primer sequences. PCR was conducted in two steps: in the first PCR reaction PCR primers were linked to tags and partial 454-sequencing adaptors A and B; in the second reaction the full 454-adaptors A and B served as PCR primers, completing the full 454-adaptor+tag+PCR primer construct. Thus, the composite forward primer in the first PCR reaction was: 5′ GTCTCCGACTCAG (NNNNNNNN) *TTGGAGGGCAAGTCTGGTGCC* 3′; and the reverse primer: 5′ TTGGCAGTCTCAG (NNNNNNNN) *GAACCCAAACACTTTGGTTTCC* 3′, where the A and B adaptors are underlined, the bar-code is indicated by N-s in parentheses, and the specific primers NS31 and AML2 are shown in italics. The 10 × diluted product of the first PCR reaction was used in the second PCR with primers A (5 ′-CCATCTCATCCCTGCGTGTCTCCGACTCAG-3′) and B (5 ′-CCTATCCCCTGTGTGCCTTGGCAGTCTCAG-3′). The reaction mix contained 5 µl of Qiagen HotStarTaq Master Mix (Qiagen Gmbh, Germany), 0.2 µM of each primer and 1 µl of template DNA, in a total volume of 10 µl. The reactions were run on a Thermal cycler 2720 (Applied Biosystems) under the following conditions: 95°C for 15 min; five cycles of 42°C for 30 s, 72°C for 90 s, 92°C for 45 s; 35 (first PCR) or 20 (second PCR) cycles of 65°C for 30 s, 72°C for 90 s, 92°C for 45 s; followed by 65°C for 30 s and 72°C for 10 min. PCR products were separated by electrophoresis through a 1.5% agarose gel in 0.5 × TBE, and the PCR products were purified from the gel using the Qiagen QIAquick Gel Extraction kit (Qiagen Gmbh, Germany) and further purified with Agencourt® AMPure® XP PCR purification system (Agencourt Bioscience Co., Beverly, MA, USA). A total of 250 ng of the resulting DNA mix was sequenced on a Genome Sequencer FLX System, using Titanium Series reagents (Roche Applied Science) at GATC Biotech (Constanz, Germany).

### Bioinformatical Analyses

Sequence reads were included in subsequent analyses only if they carried the correct forward primer sequence, a barcode matching one used in this study and were ≥170 nucleotides in length (excluding the barcode and primer sequences). This yielded 231 274 reads, varying between 170 and 557 nucleotides in length (median = 382) (Figure S1). In parallel, the presence in the raw data of chimeric sequences was investigated using UCHIME [55] in reference database mode (using the Maarj*AM* database). No reads returned a chimera score >3.2. Since a suitable threshold score for chimera detection may be as high as 5 (the range 0.1–5 is suggested by [55]) or even higher (10 [56], [57]), and for inclusion in our analysis reads were required to closely match a reference sequence over the full length, we did not remove any reads from our analysis and assumed the influence of chimeras to be low.

We used a closed reference OTU picking approach (*sensu* [58]; OTUs - operational taxonomic units – are entities used for taxonomic comparison; the concept may be applied to different taxonomic levels and hierarchies) for taxonomic assignment of the obtained reads. Thus, after stripping the barcode and primer sequences, we used the Maarj*AM* database [17] of published Glomeromycota SSU rRNA gene sequences to identify obtained reads. The Maarj*AM* database contains representative sequences covering the NS31/AML2 amplicon from published environmental Glomeromycota sequence groups and known taxa. As of February 2012 it contained a total of 3 191 records that could be associated with SSU sequence-based taxa, or so-called virtual taxa (VT cf. [22]). Sequence reads were assigned to VT (i.e. VT were the OTUs used in our analysis) by conducting a BLAST search (soft masking with the DUST filter) against the Maarj*AM* database with the following criteria required for a match: sequence similarity ≥97%; the alignment length not differing from the length of the shorter of the query (pyrosequencing read) and subject (reference database sequence) sequences by more than 10 nucleotides; and a BLAST e-value <1e-50. Where a read received multiple hits, the best hit on the basis of the BLAST score was selected. While this approach is only as comprehensive as the database against which reads are matched, it has the benefit of recording stable OTU identifiers and of representing a strict quality filter, since sequences that diverge significantly from reference sequences, e.g., non target organisms or chimeric sequences, are unlikely to be recorded [58]. A total of 13 346 pyrosequencing reads were assigned to a recorded AM fungal VT. Samples yielding <10 hits and VT that were singletons among Järvselja samples from the entire run were removed (the run included other AMF samples from the same site that are not presented here), leaving a data matrix consisting of 44 samples (i.e., 81% of collected samples) and 13 320 hits. A set of representative sequence reads (listed in Table S1) has been deposited in the EMBL nucleotide collection. We investigated those reads that BLAST did not match against the Maarj*AM* database by conducting a further BLAST search against the INSD non redundant database using the same parameters, other than relaxing the similarity threshold to 90%. Read filtering, removal of primer and barcode sequences and parsing of BLAST output was carried out using a series of Python and Java scripts developed in house.

Microbial diversity studies employing next generation sequencing are susceptible to bias if differences in read length influence taxonomic assignment [59]. We investigated the robustness of our approach by selecting reads containing >400 nucleotides and recalculating the best BLAST hits (as described above) after trimming the read set (the 3′-end of reads was trimmed) to six different lengths: 400, 350, 300, 250, 200 and 170 nucleotides (170 representing the minimum read length we considered to be adequate quality). The correspondence of hits and mean within-sample similarity between the different length sets was calculated.

### Statistical Methods

Sampling efficacy was assessed with rarefaction analysis of data subsets, using the function rarefy() from the R package vegan [60]. Thereafter, the data matrix was normalised by dividing cells by row totals (i.e. calculating the proportional composition of samples). PERMANOVA (using the function adonis() from vegan) was used to partition variance in AMF community composition in relation to spatial and seasonal replication. Bray-Curtis dissimilarity (BC) was used as a measure of distance between pairs of AMF communities:


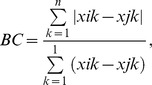


where *i* and *j* are different samples, k are the different virtual taxa, and x is the proportional composition for a given sample and taxon. The significance of factors was assessed through comparison with 999 randomised data sets (sample labels permuted).The possibility of significant effects arising due to differences in multivariate dispersion rather than compositional change was excluded on the basis of beta diversity measurements within and between groups (using function betadisper() from vegan; in relation to spatial replication: F_3,23_ = 1.47, P = 0.25).

The relationship between AMF community similarity and spatial or temporal distance as a continuous variable was investigated using a distance decay of similarity approach [61]. Thus, similarity (1-BC) between all pairs of AMF communities (i.e., between all samples) was modeled following logit-transformation of the dependent variable, which is theoretically bounded between 0 and 1. For both distance measures a model containing a log-transformed independent variable did not outperform one where the independent variable was untransformed (AIC; spatial data, untransformed  = 125.55, log-transformed  = 125.35; seasonal data, untransformed  = 152.25, log-transformed  = 152.22; and inspection of plots (Figure S2) supported the suitability of the untransformed model); slope coefficients (β) from untransformed models were therefore considered as an adequate measure of change in community similarity. The significance of the decay was assessed by comparing the empirical β with analogous parameters produced following 999 randomisations of the data set (sample labels permuted).

Diversity calculations based on next generation sequencing results can be sensitive to between-sample variation in the numbers of reads, and some authors recommend randomly thinning data matrices so that every sample is represented by the same number of reads (i.e., equalised to the size of the smallest sample in the original matrix; [59]). Therefore, we repeated the main analyses (PERMANOVA and distance decay) using 1000 data matrices where read counts per sample were equalised to the level of the lowest count (13), each time randomly selecting among the recorded hits. To reduce calculation time, randomisation was not used to assess the significance of distance decay calculations; rather the slope coefficients from these models were recorded, and their distribution with respect to 0 assessed.

## Results

### AMF Diversity in Soil

**Figure 2:**
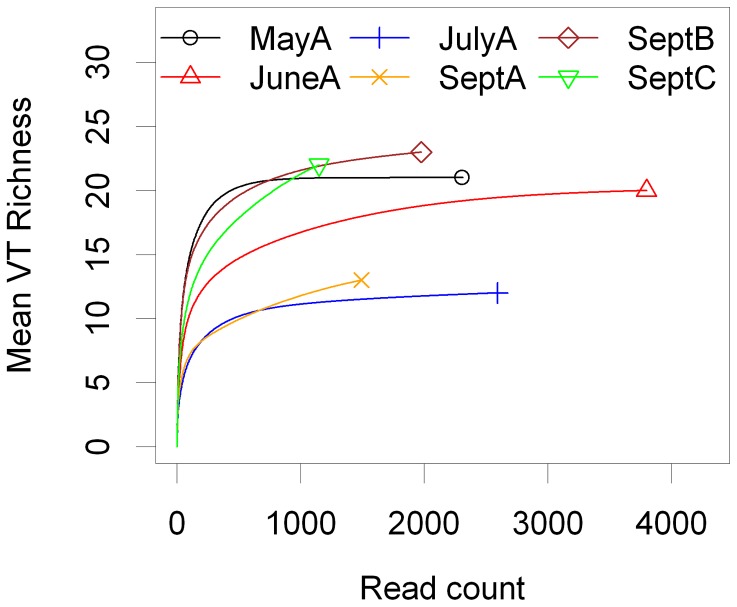
Rarefaction analysis of soil AMF samples from Järvselja forest reserve. Mean VT richness is estimated in relation to the number of reads analysed for subsets of the data representing soil samples collected in different months and from different plots.

A total of 13 320 pryosequencing reads were assigned to 37 AMF VT (Table 1; Table S2). Rarefaction analysis suggested that sampling was generally sufficient to produce asymptotic estimates of VT richness per plot and season; though estimated richness in Plot C (September) was not asymptotic (Figure 2). Of the reads that did not get a hit against Maarj*AM*, 73% got a hit (similarity ≥90%) against the INSD nucleotide collection. These hits mostly represented metazoa (94%), while fungi, including Glomeromycota, represented <1%.

**Table 1:** Sampling of AMF communities in soil at Järvselja forest reserve.

	N samples	Mean reads (range)	N VT
Plot A May	8	289 (81–625)	21
June	9	422 (36–1338)	20
July	6	432 (18–1986)	12
Sept	4	373 (32–1078)	13
Plot B Sept	8	247 (50–880)	23
Plot C Sept	9	128 (13–525)	22

N samples shows the number of samples that yielded over 10 BLAST hits against the Maarj*AM* database of AMF SSU diversity; note that 9 samples were collected from each soil plot (9 per season in plot A). The mean number and range of reads per sample is presented, as is the total number of AMF OTUs (VT, i.e., virtual taxa) per soil plot in each season.

### Spatial Variation in Soil AMF Communities

**Figure 3:**
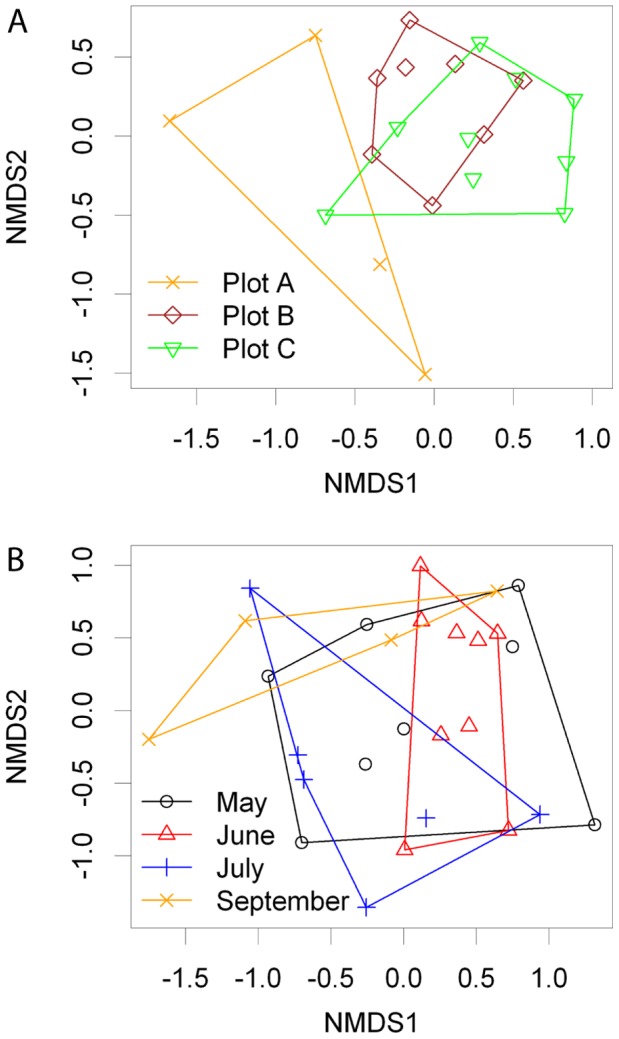
Figure 3. Two-dimensional non-metric multi-dimensional scaling (NMDS) plots of variation in soil AMF community composition. A) in three spatially distinct 10×10 m plots (A–C) in September; and B) in plot A in May, June, July and September.

**Figure 4:**
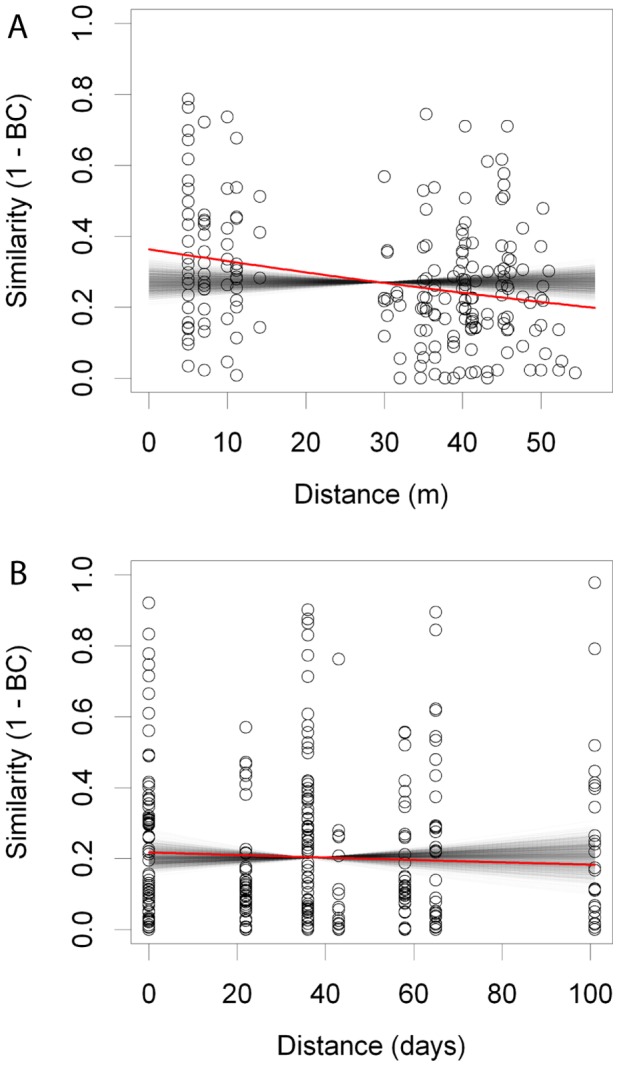
Change in soil AMF community similarity as a function of spatial and temporal distance. A) spatial - soil samples from 10×10 m plots in September and; B) temporal - soil samples from plot A in May, June, July and September. The data points show pairwise similarity estimates between all samples; the bold red line shows the relationship between similarity and distance in the real data; the faint black lines show the same relationship in 1000 randomised data matrices. Note that the x axis does not represent direction in space or time; thus greater values denote the greatest distance between samples – e.g. between May and September – and not the timing or location of samples *per se*. The slope of the real model was steeper than the randomised set for the spatial model (P<0.01) but not the temporal model (P = 0.57).

The taxonomic richness of AMF communities in soil samples did not differ significantly between plots (plot A: mean  = 5.00, s.e. = 1.08; plot B: mean  = 8.75, s.e.  = 1.41; plot C: mean  = 6.78, s.e. = 0.62; Poisson GLM χ^2^ = 0.83, P = 0.66); however, there were significant differences in community composition between plots (Table 2; Figure 3a). Within plot A, sample identity (1–9) did not explain a significant proportion of the variation in community composition between the repeated measures taken in different seasons (Table 2). Pairwise similarity of AMF communities decreased as a linear function of the spatial distance between samples (slope coefficient on logit scale β = −0.011); and randomisation analysis indicated that this decrease was significantly greater than expected under the null hypothesis of no distance effect (P<0.01; Figure 4a).

**Table 2:** Variation in the AMF communities present in soil at Järvselja forest reserve.

Model/Parameter	DF	SS	MS	Pseudo F	R^2^	*P*
**Seasonal**						
Season	3	1.35	0.45	1.34	0.15	0.17
SampleID	8	2.41	0.31	0.89	0.27	0.71
Residuals	15	5.06	0.34		0.57	
Total	26	8.83				
**Spatial**						
Plot	2	1.17	0.58	2.36	0.21	0.001
Residuals	18	4.46	0.25		0.84	
Total	20	5.63				

The results of PERMANOVA analysis are shown. The seasonal model describes variation between sampling month (May, June, July and September) and sample location (SampleID) in Plot A; the spatial model describes variation between Plots A, B and C in September. In the seasonal model, the significance of explanatory variables was not sensitive to their order in the model.

### Seasonal Variation in Soil AMF Communities

VT richness did not vary significantly in relation to season (May: mean = 5.88, s.e. = 1.14; June: mean = 6.89, s.e. = 0.75; July: mean = 4.33, s.e. = 1.02; September: mean = 5.00, s.e. = 1.08; Poisson GLM χ^2^ = 4.63, P = 0.20), and there was no significant change in community composition during the sampling period (Table 2; Figure 3b). Neither was there evidence of temporal decay in community similarity (slope coefficient on logit scale β = −0.00088, P = 0.57, Figure 4b). Thus, temporally close samples were on average as similar to each other as temporally distant samples.

### Pattern Robustness

We trimmed a set of long reads to different lengths, including the minimum length we considered to contain adequate taxonomic signal in this study (170 nucleotides), and repeated taxonomic assignment of all read sets. The taxonomic identity of reads and profile of communities remained similar between the longest set (400 nucleotides) and all other sets (Table 3).

**Table 3:** Taxonomic assignment using read sets trimmed to different lengths.

	Trimmed read length (nucleotides)				
	170	200	250	300	350
Identical hits N/%	4 371/96.4	4 341/95.7	4 439/97.9	4 480/98.8	4 495/99.1
Identical hits N/%; no hits removed	4 305/98.5	4 271/98.5	4 361/99.6	4 395/99.9	4 409/99.9
Richness N/% of 400 set	29/96.7	29/96.7	28/93.3	29/96.7	29/96.7
Mean sample-wise similarity to 400 set: BC dissimilarity/Pearson’s r	0.034/0.983	0.041/0.981	0.0193/0.998	0.009/0.999	0.004/0.999

Results for six set lengths between 170 (the minimum acceptable read length considered in this study) and 350 nucleotides are compared with the set of 400 nucleotide reads; thus the cells of the table always represent a comparison with the characteristics of the 400 nucleotide set. ‘Identical hits’ shows the number and percentage of reads getting identical hits irrespective of read length; ‘Identical hits; no hits removed’ shows the same calculations but omitting any read that did not get a hit in either of the compared sets; ‘Richness’ shows the total number of VT and the richness as a percentage of that in the 400 nucleotide set; ‘Mean sample-wise similarity’ was a comparison of the community composition of the same sample in the 400 nucleotide set and in the shorter trimmed sets. This was calculated using two metrics: Bray-Curtis dissimilarity and Pearson’s correlation coefficient. Samples containing fewer than 10 reads were removed from the data set, leaving 42 samples and 4 508 reads. VT abundance was normalised (converted to proportions) prior to similarity calculations.

Reanalysis of 1000 data matrices randomly thinned to contain 13 reads per sample produced the following results: (i) the effect of plot in the spatial PERMANOVA remained significant in 95% of thinned matrices; (ii) the effects of season and sample in the seasonal PERMANOVA became significant in fewer than 2% of thinned matrices; (iii) the slope of the spatial distance decay remained positive (i.e. becoming more dissimilar) in 100% of thinned matrices; (iv) the slopes of the seasonal distance decay were neither consistently positive nor negative in thinned matrices (>0∶90%; <0∶10%). Moreover, VT richness was not dependent on sample size (Figure S3).

## Discussion

AMF can be detected *in planta* and in soil using DNA-based methods [62], [63]. Next generation sequencing (NGS) techniques allow amplicons to be recovered from environmental samples with a depth that is orders of magnitude greater than was previously possible. This can generate higher richness estimates and potentially more comprehensive data about microbial community composition than obtained using earlier methods [22]. Consequently, it is becoming increasingly possible to answer questions about the distribution and dynamics of unculturable microorganisms in natural ecosystems. However, the lengths of reads returned by NGS vary, and in bar-coding studies such as this one, the size of libraries inevitably differ, which can potentially bias between-sample diversity comparisons [59], [64], [65]. Here, we show that a BLAST-based closed reference OTU picking approach (*sensu* [58]) to taxonomic assignment is robust to read length variation when using the NS31-AML2 amplicon and a minimum read length threshold of 170 nucleotides. We also found that our diversity analyses were robust to heterogeneity in read counts per sample, assuming a minimum of 10 reads per sample.

We recorded a total of 37 AMF taxa from the Järvselja natural forest study site. There are few comparable works carried out on soil samples against which to judge this figure, but data that are available fall into a similar range: pyrosequencing approaches have recorded 27 OTUs from soils in Sardinian cork-oak scrubland [28] and 33 from arable land in Canada [37]. However, recent studies of root AMF communities have revealed considerably higher fungal richness from forest environments: 70 OTUs (using a slightly finer resolution than the VT classification) at a woodland site in the United Kingdom [20]; and 57 VT in a different Estonian forest study area (Koeru; [22]–[24]). Targeting DNA extracted from soil provides an assessment of the AMF taxa that are actively forming mycorrhizae, plus taxa present in the form of spores - though calculations by Hempel et al. [25] indicate that the contribution of spores should be low. The molecular diversity of soil AMF might therefore be expected to represent a species pool of which *in planta* AMF communities would constitute a fraction, and this is supported by evidence of higher AMF richness in soil than root samples in some studies [31], [38], [40], [42]. However, the opposite pattern or no difference has also been reported [30], [33], [36], [39]. While these latter findings and a*d hoc* comparison of our results with root studies from forest sites indicate higher root-based diversity, it is notable that a low proportion (5.8%) of the reads meeting quality control criteria derived from our soil samples represented AMF. For comparison, in the forest plant root studies described above, AMF represented 70.6% ([22]; percentage of reads meeting quality control criteria) and 76.2% ([20]; percentage of all reads) of reads. This matches previous findings indicating low recovery of Glomeromycota SSU DNA from rhizosphere or soil samples compared with clean root samples [28], [66], [67]. This possibly reflects the lower proportion of AMF DNA in the total DNA pool in soil as compared to colonised roots, and the tendency of the chosen primers to amplify non-AMF organisms to some degree. It should be noted however that primer combinations that would amplify all AMF and exclude all other organisms have not yet been identified, though several options have been proposed [68], [69]. Besides AMF sequence yield per sample, lower template abundance might also affect richness estimation [65], [70], rendering a direct comparison between soil and root AMF community fractions a complicated task.

Our results revealed spatial variation in the composition of the forest soil AMF community. Community similarity decreased, albeit modestly, as a linear function of distance up to approximately 50 m, while partitioning of variance in AMF community similarity indicated systematic variation between plots located 30 m from one another (the effect of Plot in the spatial model in Table 2), but none within a single 10×10 m sampling plot (the effect of SampleID in the seasonal model in Table 2). It should be noted, however, that the within- and between-plot comparisons had different temporal scopes and different power to detect significant effects. Thus, it would be inappropriate to discount the possibility of a spatial effect operating at scales <10 m. Though study plots were located in an area of forest that contained no clear environmental gradients, variation at the between-plot scale might be related to heterogeneity in vegetation and soil conditions. The understorey vegetation of Järvselja old growth forest is patchy and varies more in space than that of younger successional forests [50], [51]. Such patchiness of vegetation could drive the spatial structure of soil AMF communities we observed through a host plant effect on AMF diversity and composition [3], [22]; at the same time, structure in AMF communities may contribute to highly variable germination conditions for plant seedlings [26], generating linked spatial assortment of plant and AMF communities. Our results are in accordance with studies that have found AMF spores to be patchily distributed at multiple scales [71]–[73]. Studies addressing AMF communities in plant roots have also reported spatial variation [46]. As far as we are aware, the only DNA-based investigation of spatial variation in soil AMF communities comes from Mummey and Rillig [26], who used the LSU rRNA gene and demonstrated that AMF abundance and community composition in soil can be definably spatially structured at scales of <1 m. Our results indicate that structure in soil AMF communities, and presumably any consequent influence on wider ecological processes, may also operate at ‘local’ scales greater than 1 m.

Studies using plant root samples have revealed seasonality of root colonisation by individual members of the AMF community [19], [20], [23], [48], [49]. Spore community composition has also been shown to be seasonally dynamic [73], [74]. Our results showed that the taxon composition of the AMF community in soil (i.e. spores and extraradical mycelia) at Järvselja was relatively stable during a *c*. 4 month period corresponding to the majority of the vegetation growth period. Partitioning variance in community composition revealed no significant effect of season and indicated that far more variation was apparent within- rather than between- seasons. In this sense our results support the concept of natural ecosystems containing a relatively constant pool of AMF taxa in the soil, from which plant-AMF interactions form and disband during the year. Such dynamic interactions may reflect sorting of suitable partners throughout the season or changing nutrient requirements and availabilities acting upon both plant and fungal partners.

The results of this study indicate some potentially useful directions for future research. In particular, the generality of the seasonal result - a lack of systematic soil AMF community change during the growing season - could be determined by investigating seasonal trends in other systems. It would be particularly useful to investigate seasonality in relation to variation in environmental factors (e.g., temperature, moisture and disturbance), and in host plant community characteristics, such as phenology. Such information would have great value in establishing the importance of seasonal coverage for representative survey design and the comparability of soil AMF community data collected at different time points. The spatial pattern identified in this study also raises further questions. The importance of biotic and edaphic variables in driving spatial variation in soil AMF community structure remains to be determined. It would for example be valuable to measure covariation in plant and AMF community composition, considering both AMF in soil and in plant roots.

## Supporting Information

Figure S1 **Decay in soil AMF community similarity in relation to A) spatial or B) temporal distance.** Plots show a comparison of model fits using an untransformed (green line) or log-transformed (blue line) independent variable. AIC values: spatial model, untransformed  = 125.55, log-transformed  = 125.35; seasonal model, untransformed  = 152.25, log-transformed  = 152.22.
(TIF)

Figure S2 **Distribution of pyrosequencing read lengths.**
(TIF)

Figure S3 **Relationship between between number of reads per sample (sample size) and VT richness.**
(TIF)

Table S1 **Sequence reads deposited in the EMBL nucleotide collection.**
(XLS)

Table S2 **AMF VT detected in soil samples.**
(XLS)
