# Supplementary figures and images for: Communities of Arbuscular Mycorrhizal Fungi Detected in Forest Soil Are Spatially Heterogeneous but Do Not Vary throughout the Growing Season
Source: PLoS One. 2012 Aug 7;7(8):e41938. doi: 10.1371/journal.pone.0041938 (PMC3413688; doi:10.1371/journal.pone.0041938)

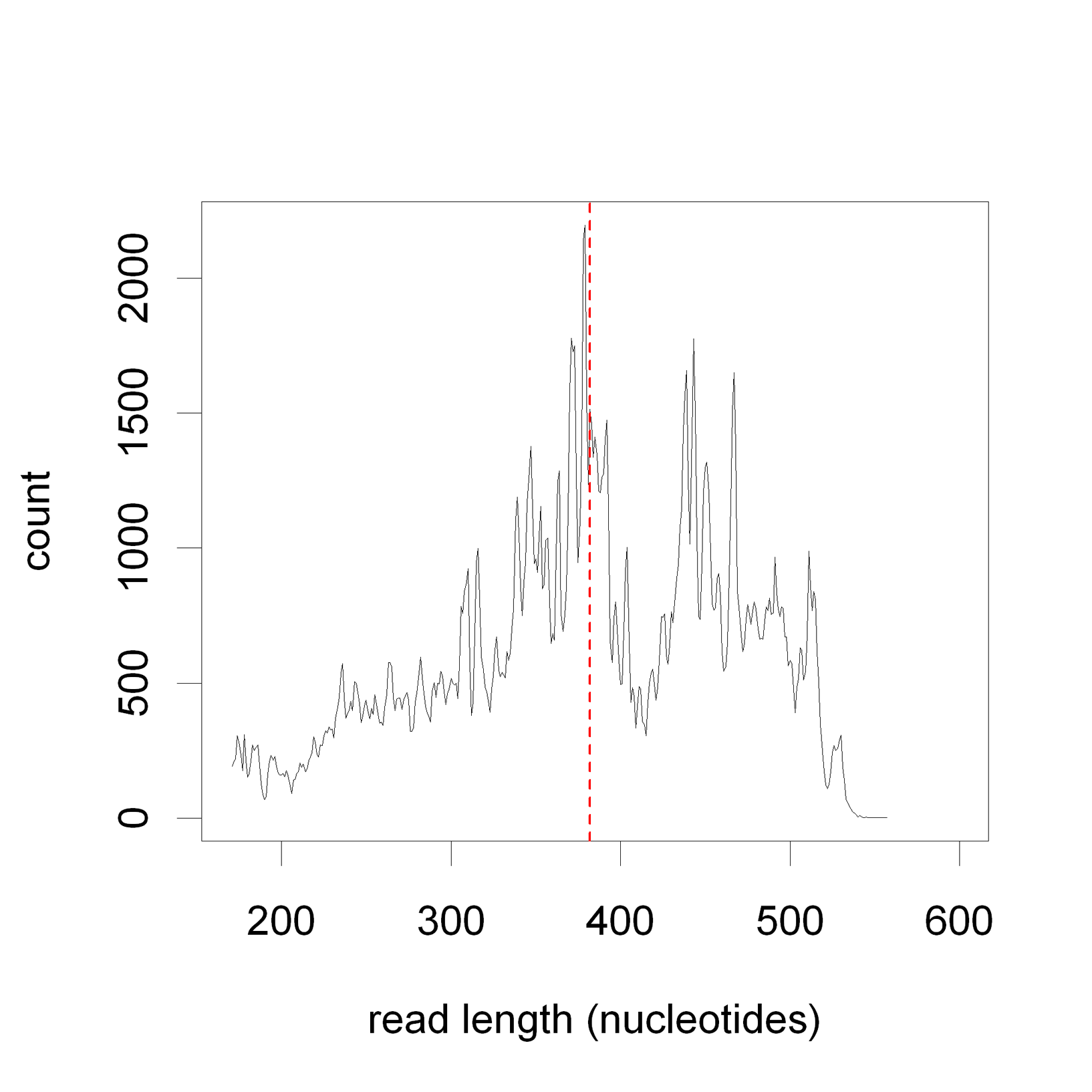

Supplement: Figure S1 — Decay in soil AMF community similarity in relation to A) spatial or B) temporal distance. Plots show a comparison of model fits using an untransformed (green line) or log-transformed (blue line) independent variable. AIC values: spatial model, untransformed = 125.55, log-transformed = 125.35; seasonal model, untransformed = 152.25, log-transformed = 152.22. (TIF) [file pone.0041938.s001.tif]

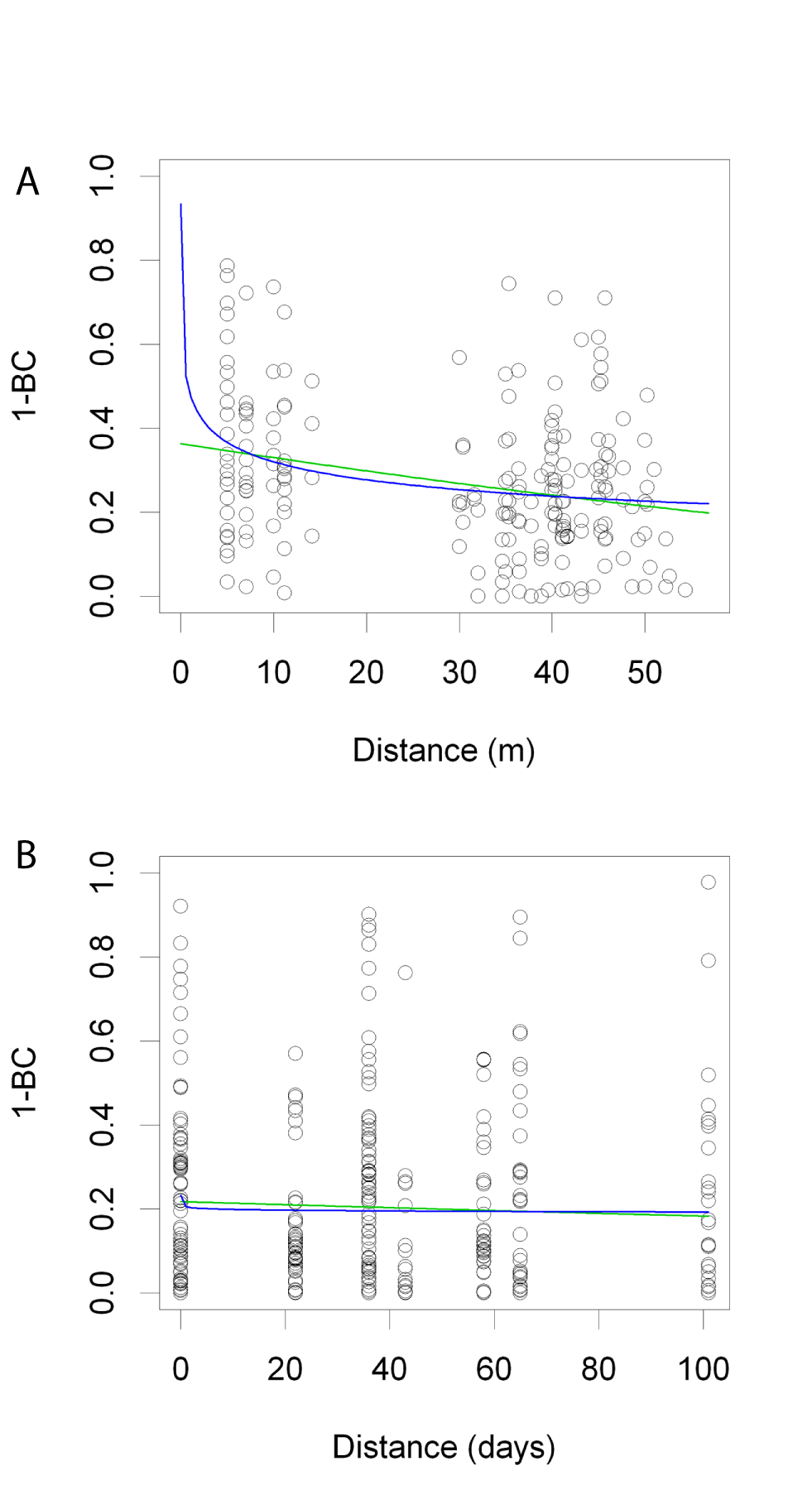

Supplement: Figure S2 — Distribution of pyrosequencing read lengths. (TIF) [file pone.0041938.s002.tif]

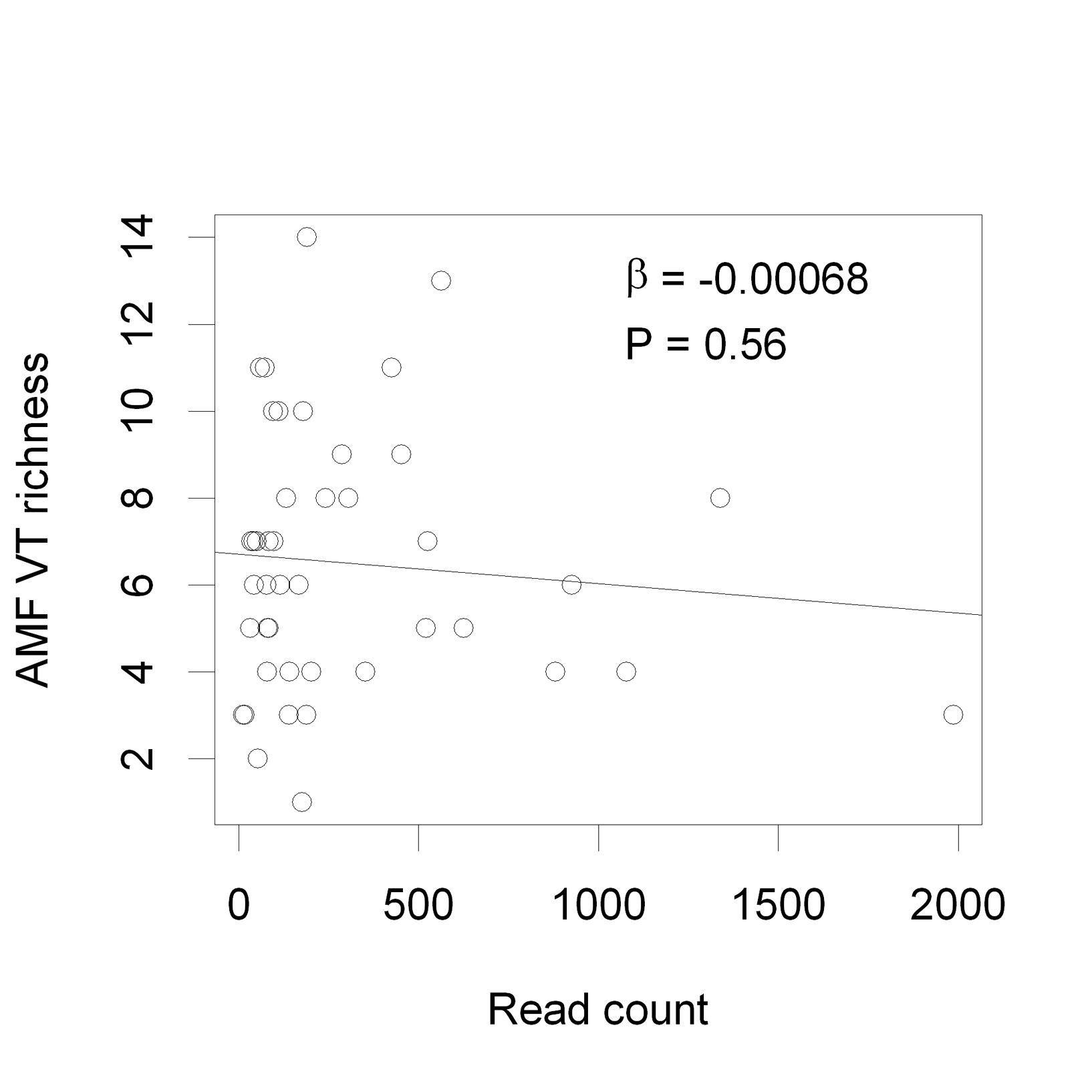

Supplement: Figure S3 — Relationship between between number of reads per sample (sample size) and VT richness. (TIF) [file pone.0041938.s003.tif]
